# Mechanical Properties of Cervical Spinal Cord in Neonatal Piglet: *In Vitro*

**DOI:** 10.31487/j.nnb.2020.02.08

**Published:** 2020-05-28

**Authors:** Anita Singh, Rachel Magee, Sriram Balasubramanian

**Affiliations:** 1 Widener University, School of Engineering, Chester, Pennsylvania, USA; 2 Drexel University, School of Biomedical Engineering, Science and Health Systems, Philadelphia, Pennsylvania, USA

**Keywords:** Neonatal, spinal cord, stretch, injury, biomechanical properties

## Abstract

The response of neonatal spinal cord tissue to tensile loading is not well-studied. In this study, isolated fresh neonatal cervical spinal cord samples, obtained from twelve 2-4 days old piglets, were tested in uniaxial tension at a rate of 500 mm/min until failure. Maximum load, maximum stress, percentage strain at maximum stress and modulus of elasticity were reported to be 14.6±3.4 N, 0.34±0.11 MPa, 29.3±5.4% and 1.52±0.8 MPa, respectively. These data can help understand the biomechanical behavior of the spinal cord in neonates and can be further used in computational modeling to understand injury mechanisms better and help develop injury prevention strategies.

## Introduction

Neonatal spinal cord injuries that occur *in utero* or during a complicated delivery result in significant long-term deficits in sensory and motor functions [1]. Understanding the threshold for neonatal spinal cord injury is critical not only for a better understanding of the injury mechanism but also for developing strategies that can lead to injury prevention. Since neonatal injuries cannot be studied in humans, for ethical reasons, alternatives such as physical and computational models that simulate injury scenarios are commonly employed. The validity of such models heavily relies on an understanding of the biomechanical responses of the neonatal spinal cord when subjected to stretch.

While several studies have investigated the mechanical properties of the adult spinal cord in both adult human and other animals, the biomechanical threshold values for neonatal spinal cord tissue is not available. Clarke et al. (2009) reported the stress-relaxation responses of the spinal cord in young and adult rats [2]. While their study confirmed a higher initial severity of spinal cord injury in younger animals when compared to adults, it failed to report failure values for the neonatal spinal cord. Lack of data on biomechanical responses of the neonatal spinal cord warrants studies that can fill this gap, especially using a neonatal large animal model with anatomical similarities to humans.

The objective of the current study was to measure the response of the neonatal piglet spinal cord when subjected to uniaxial stretch. We obtained load displacement responses from the neonatal spinal cord of piglets when subjected to tensile loading at a 500 mm/min rate until failure. Maximum load, maximum stress, strain at maximum stress, and modulus of elasticity were reported. These data can be applied to enhance the human-like response of the existing computational models of neonatal spinal cord.

## Methods and Materials

### I Tissue Collection

A total of 12 cervico-thoracic spinal cord segments (C3-T2) obtained from twelve, immediately post-partum, normal neonatal piglets (3-5 days old) were used in this *in vitro* study. These freshly harvested spinal cord samples were preserved in phosphate-buffered saline until testing, which was performed within two hours after tissue removal.

### II Mechanical Test Setup

**Figure 1:**
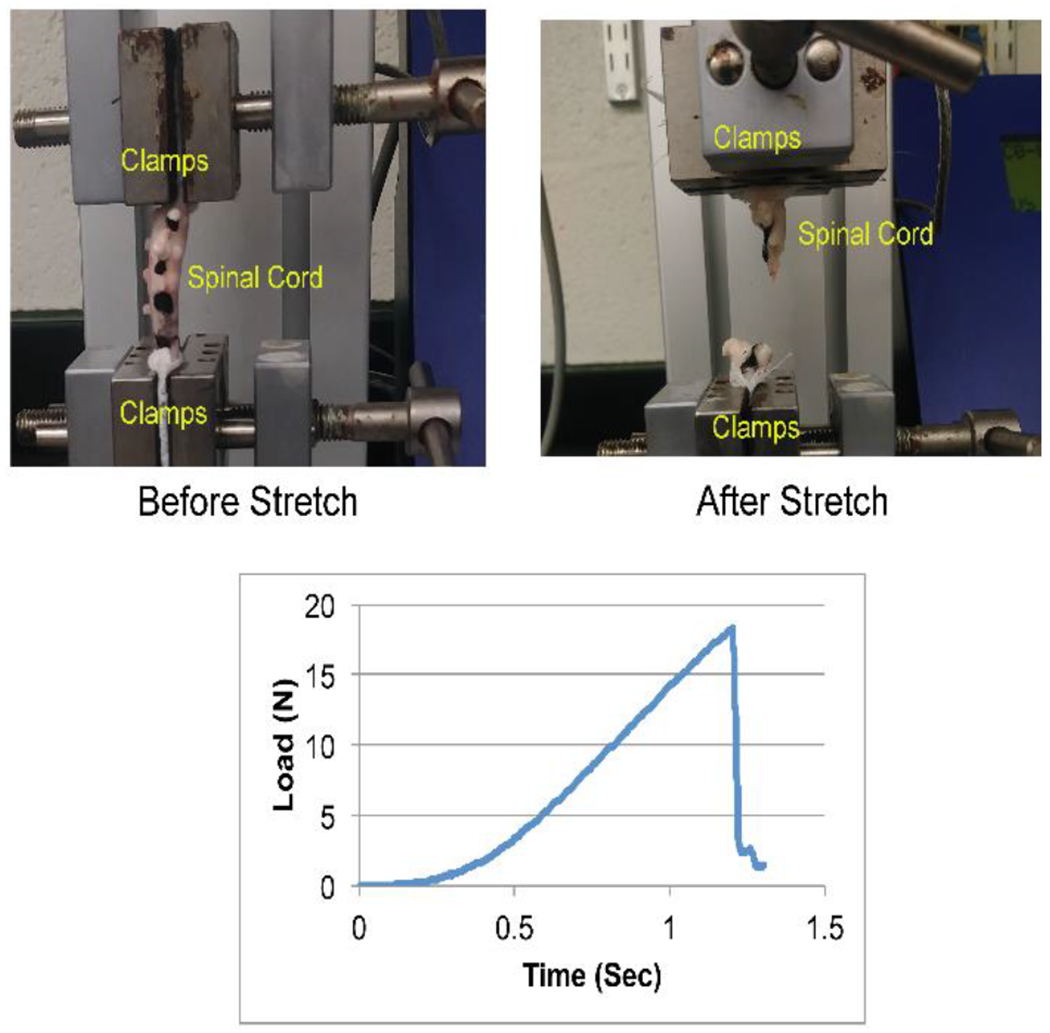
Mechanical testing setup (top) and exemplar load-time response (bottom) during the tensile loading of a neonatal piglet spinal cord.

A tensile testing machine (eXpert 7600, ADMET Inc., MA) was used to stretch the spinal cord samples (Figure 1). Each sample was anchored to the testing setup using two clamps such that one end of the spinal cord sample was attached to the fixed end of the machine, and the other end to the actuator via a 50 N load cell (Figure 1).

### III Camera System Setup

A high-speed video camera, Basler acA640-120uc camera (Basler, Pennsylvania), which collected data at 120 fps was positioned in front of the test specimen to capture the movement of fiducial markers placed on the spinal cord tissue during the pull. Displacements of these markers were tracked for calculating strain.

### IV Tensile Testing Procedure

A digital microscope (5X; Digital VHX Microscope, NJ) was used to obtain images of the harvested spinal cord tissue. A 2 mm ruler (Leitz, Ernst-Leitz-Wetzlar GmbH, Germany) was co-imaged at the same magnification to measure the tissue diameter. The two clamps were initially set at a distance of 50-100 mm (depending on the initial length of the tissue), and the testing sample was then clamped with no initial tension prior to stretch. The actuator displacement rate was controlled by a built-in GaugeSafe software (ADMET Inc., MA), which applied stretch at a rate of 500 mm/min until complete tissue failure. Time, load, and displacement data were acquired at a sampling rate of 1000 Hz during the entire test duration. After the completion of the experiment, the failure site was recorded (example: at or closer to actuator clamp, at or closer to stationary clamp, or mid-length of the tissue). Finally, the clamps were checked for the presence of tissue. No tissue in a clamp implied that the sample had completely slipped, and data from those experiments were discarded.

### V Data Analysis

Load data were converted to nominal stresses (i.e., load/original cross-sectional area of the tissue, assuming a circular tissue cross-section). Displacement data, obtained by tracking the displacements of markers placed on the tissue, were used to calculate tensile strain (i.e., Strain (%) = [(L_f_−L_i_)/L_i_] x 100; where L_i_ is initial tissue length, L_f_ is final tissue length). The load–displacement and stress–strain curves were plotted, and the maximum load, maximum stress, strain at the point of maximum stress, and Young’s Modulus (E; the slope of stress–strain curve after toe region and below the proportional limit) were determined. The video data were also used to track changes in the structural integrity of the tested samples [3]. As load, actuator displacement, and video data were recorded synchronously, the relationships between these datasets could be characterized.

## Results

Out of 12 tested spinal cord tissue samples, two samples slipped during tensile testing and were excluded from data analysis. In the remaining ten samples, failure was observed over the entire length of the tissue. In 80% of those cases, rupture occurred at mid-length of the tissue, as shown in (Figure 1). In the remaining 20% of the cases, the rupture was observed closer to the actuator clamp side. The reported average and standard deviation values for maximum load, maximum stress, strain at maximum stress and E are summarized in (Table 1).

## Discussion

Mechanical forces induced during traumatic scenarios can cause permanent damage to the spinal cord. The available literature on the biomechanical responses of the spinal cord that are primarily from adult small animals and humans, exhibit large discrepancies in their findings [4–7]. Tissue processing (e.g., fixed, unfixed tissue), methodological differences in measuring stretch and differences in species contributed to variations in the available literature. Furthermore, no study has used fresh spinal cord tissue from neonates. Data obtained from this study is the first to offer detailed mechanical responses from neonatal spinal cord using a neonate large animal model that is clinically relevant.

While biomechanical data from human neonate spinal cord tissue would be ideal, it is difficult to obtain. Using neonatal piglet (large animal) model, which has close anatomical similarities to human and have been previously used to study neonatal injuries, can serve as a good surrogate to understanding biomechanical responses in human neonates [8]. When compared to previously reported maximum load (32-56 N) and modulus of elasticity (0.2-0.4 MPa) values from adult pig spinal cord, corresponding data from the current study on neonatal piglets (maximum load: 14.6±3.4 N and modulus of elasticity: 1.52±0.8 MPa) confirm the effect of age on mechanical responses of the spinal cord [2]. Future studies could explore the factors that lead to these differences.

In summary, the current study is the first to report biomechanical properties of fresh neonatal piglet spinal cord. These data can be used to develop an improved computational model of pediatric spinal cord that can accurately illustrate the contributions of predisposing risk factors for spinal cord injury in neonates, thereby advancing the science of neonatal care.

## Figures and Tables

**Table 1:** Mechanical responses (Average ± Standard Deviation) from *in vitro* tensile testing of neonatal piglet spinal cords (n=10).

Parameters	Values
**Maximum Load (N)**	14.6±3.4
**Maximum Stress (MPa)**	0.34±0.11
**Strain (%) at Maximum Stress**	29.3±5.4
**E (MPa)**	1.52±0.8
